# Angiomatouse meningioma with intracerebral hemorrhage: A case report and literature review

**DOI:** 10.1016/j.radcr.2024.06.081

**Published:** 2024-08-23

**Authors:** Ibtisam Al-Huthali, Sarah Alem, Abdullah Darwish, Zaina Brinji, Basem Bahakeem

**Affiliations:** a Department of Radiology, King Abdullah Medical City, Makkah, Saudi Arabia; b Department of Internal Medicine, Umm Al-Qura University, Makkah, Saudi Arabia

**Keywords:** Angiomatous, Meningioma, Intracranial, Hemorrhage

## Abstract

Intracranial angiomatous meningioma (AM) is a rare meningioma subtype. Hemorrhage is a rare meningioma-related complication that can occur spontaneously. Therefore, this specific medical condition is rarely described in the literature. We describe a rare case of a 65 year's old female with repeated focal left upper limb epileptic attacks and no history or evidence of neoplasms, trauma, or coagulopathy. Magnetic resonance imaging and computed tomography (MRI and CT, respectively) are early and accurate diagnostic tools that reveal an intracerebral hemorrhage from a right occipital meningioma. She was treated by surgical resection. The diagnosis was confirmed based on the pathological analysis of the resected tumor that was reported as an AM.

## Introduction

Meningiomas are the most prevalent (20%-30%) primary brain tumor type with an adjusted annual incidence of about 4.5 per 100,000 individuals [1].

Angiomatous meningioma (AM) is a rare subtype of meningioma and accounts for 2.1% to 2.59% of all meningiomas [2]. Intracranial hemorrhage (ICH), also known as an intracranial bleed, is defined as bleeding within the skull [3]. Subtypes are intracerebral (intraventricular bleeds and intraparenchymal bleeds), subarachnoid, epidural, and subdural bleeds [2]. Intracerebral bleeding affects 2.5 per 10,000 people each year [2]. Spontaneous bleeding associated with intracranial neoplasms is rare and only occurs in approximately 1.4%–10% of all intracranial neoplasms [3]. In this report, we present a rare case of AM with intracerebral hemorrhage and use related literature to discuss the clinical manifestations of our case.

## Case history

**Fig. 1:**
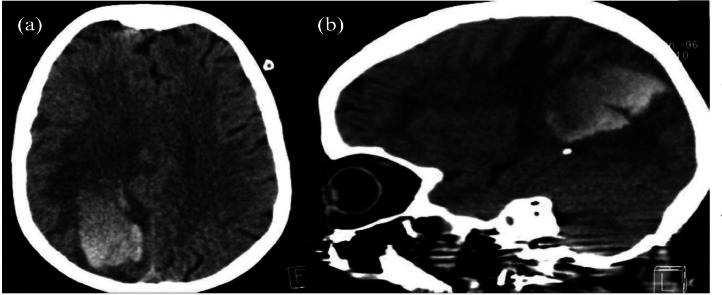
Axial (A) and sagittal (B) Nonenhanced computed tomography **(**NECT) of the brain showing right parieto-occipital intracerebral hematoma with blood-fluid level (arrow), associated with surrounding edema and mass effect (arrowhead).

**Fig. 2:**
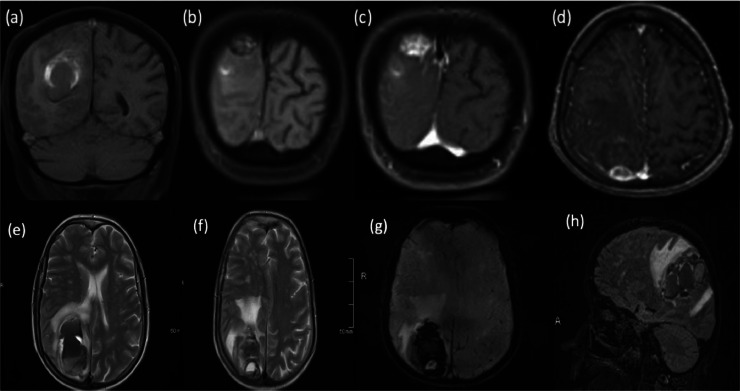
Multi-sequential magnetic resonance images (MRI) of the brain show an angiomatous meningioma (AM) at the right parieto-occipital lobe. It shows isointense signal intensity in T1WI with peripheral high signal intensity (A and B), and heterogeneous enhancement in post-contrast images (C and D) on axial T2-weighted image (T2WI) (E and F), susceptibility-weighted image (SWI) (G), and sagittal fluid-attenuated inversion recovery (FLAIR) (H) show central hemorrhagic changes and an adjacent large intra-cerebral hematoma, which depicts variable signal intensities of variable blood ages on T1, T2, and FLAIR sequences and magnetic susceptibility artifacts on SWI. Blood–fluid levels within the lesion and the intracerebral hematoma indicate active bleeding.

A 65-year-old female presented to the emergency room (ER) with a history of sudden onset of left-side weakness, confusion, and repeated focal left upper limb epileptic attacks. No history or evidence of neoplasms, trauma, or coagulopathy, and no prior imaging results were available. Non-enhanced computed tomography (NECT) of the brain showed a large right parietal-occipital intracerebral hemorrhage with associated subfalcine herniation (Fig. 1). Further evaluation with brain magnetic resonance imaging (MRI) showed a right parieto-occipital extra-axial dural-based heterogeneously enhancing lesion adjacent to the site of intracerebral hematoma (ICH) measuring 2.2×1.9×1.8 cm with central hemorrhagic changes (Fig. 2).

The patient underwent urgent surgical decompressive craniotomy and tumor resection. The entire mass and hematoma were eradicated. The histopathology evaluation revealed an AM. The patient was discharged from the hospital on the eighth postoperative day. All neurological functions recovered completely.

## Discussion

AM is a rare subgroup of meningiomas. It belongs to the World Health Organization's (WHO) grade I class and accounts for 2.1% of all meningiomas. Although AMs share similar clinical features and prognosis as benign meningiomas, the AMs have some unique characteristics, such as a relatively high male-to-female ratio when compared with meningiomas in general, more frequent peritumoral edema, and a rich supply of blood vessels in the tumor [4]. Most hemorrhage cases associated with meningiomas are extra-tumoral and subarachnoid, whereas subdural, intracerebral, and intratumoral hemorrhages have been reported less frequently [5]. Our reported case presents a rare case of AM with ICH.

The pathophysiological mechanisms of underlying spontaneous meningioma bleeding are not well understood, but several hypotheses have recently been proposed [6]. The most common theory is that a rupture within the abnormal vasculature of the tumor occurs. This diagnosis is dependent on findings, including weak thin-walled vessels in a morphological analysis or peritumoral vascular erosion by the tumor directly. Another hypothesis recommends that enlarged, tortuous feeding arteries are less resistant to blood pressure changes and therefore, are susceptible to rupture.

The clinical manifestations of an AM usually involve headache, transient loss of consciousness, seizures, nausea, vomiting, fecal incontinence, facial hypoesthesia, tinnitus and deafness, hoarseness, papilledema, unilateral limb paresis, visual impairment, sluggish corneal reflex, flat nasolabial fold, unilateral limb spasticity, ataxia, positive Babinski sign, and chewing muscle weakness [7]. Our case presented to the emergency room (ER) with a history of sudden onset of left-side weakness, confusion, and repeated focal left upper limb epileptic attacks.

Because of its high level of vascularity, AM displays intense contrast enhancement and significant peritumoral edema on CT scans. On an MRI, a dural-based mass shows homogeneous enhancement, blood flow void shadow, and peritumoral edema [8]. We mainly depended on the imaging studies for our case's primary diagnosis. In agreement with the previous explanation, the CT results were reported as a right parietooccipital intracerebral hematoma, and the MRI was a heterogeneously enhancing dural-based lesion with central hemorrhagic changes and adjacent intracerebral large hematoma.

Some factors are directly associated with peritumoral edema in meningiomas; these factors include venous obstruction, pial-meningeal anastomosis, increased capillary permeability, sexual hormones/receptors, and vascular endothelial growth factor (VEGF) secretion. The last factor is the main factor [9].

Gross total resection is the treatment of choice [6]. In our case, the patient underwent urgent surgical decompressive craniotomy and tumor resection. Based on histopathology, AM is characterized by abundant and well-formed vascular channels, sinusoids, and/or capillaries. The histopathology result of the resected tumor of our case revealed AM.

For cases with residual tumors after surgery, radiation therapy can be used to treat these patients [6]. The molecular basis of radiation therapy's effectiveness is that radiotherapy can inhibit the expression of (VEGF) and somatostatin receptors, which causes a blood vessel contraction-induced reduction in blood supply and tumor shrinkage [6]. Regarding our case, all of the mass and hematoma were eradicated, so the patient did not undergo radiotherapy.

## Conclusion

Angiomatous meningioma is a rare subgroup of meningiomas. Intracerebral hemorrhage associated with meningiomas is also rare. MRI and NECT are primary keys to diagnose with histopathology result conforming the diagnosis.

## Patient consent

I hereby confirm that written and informed consent for the publication of the case has been obtained from the patient(s) involved. The patient(s) have been fully informed about the nature and purpose of the publication, and they consent to the use of their case details in the article. I understand that the actual agreement with the patient(s) or their representative(s) should not be submitted but will be retained for my own records.
